# Multi-Sensor Arrays for Online Monitoring of Cell Dynamics in *in vitro* Studies with Choroid Plexus Epithelial Cells

**DOI:** 10.3390/s120201383

**Published:** 2012-02-01

**Authors:** Pedro Mestres-Ventura, Andrea Morguet, Soledad García Gómez de las Heras

**Affiliations:** 1 Department of Anatomy and Cell Biology, Saarland University, University Hospital, Bldg. 61, 66421 Homburg, Saar, Germany; E-Mail: wmorguet@t-online.de; 2 Department of Human Histology and Pathology, Faculty for Health Sciences, University Rey Juan Carlos I, Av. de Atenas s/n, E-28922 Alcorcón, Madrid, Spain; E-Mail: soledad.garcia@urjc.es

**Keywords:** sensor arrays, peri-cellular acidosis, cell respiration, cell adhesion, impedance, cell cultures, electron microscopy, choroid plexus, chick embryo

## Abstract

Sensors and multi-sensor arrays are the basis of new technologies for the non-label monitoring of cell activity. In this paper we show that choroid plexus cells can be cultured on silicon chips and that sensors register in real time changes in their activity, constituting an interesting experimental paradigm for cell biology and medical research. To validate the signals recorded (metabolism = peri-cellular acidification, oxygen consumption = respiration; impedance = adhesion, cell shape and motility) we performed experiments with compounds that act in a well-known way on cells, influencing these parameters. Our *in vitro* model demonstrates the advantages of multi-sensor arrays in assessment and experimental characterization of dynamic cellular events—in this case in choroid plexus functions, however with applicability to other cell types as well.

## 1. Introduction

Electrical and electrochemical sensors have enabled the development of technologies, which are very useful in obtaining data on cellular activities with minimal external intervention. These techniques can be applied without any labeling of cells, thus avoiding the possible alteration of cells under investigation. Furthermore, these techniques have paved the way for online observation, enabling continuous, uninterrupted execution of the experiment. These developments represent a considerable improvement on the more traditional so-called “end-point” techniques [1].

Pioneer devices were equipped with sensors detecting changes in cell adhesion, cell motility and cell shape, based on impedance measurements [2]. Another generation of sensors was the so-called light-addressed potentiometric sensors (LAPS), able to detect changes in peri-cellular acidosis, a phenomenon which is closely related to the state of cell metabolism [3]. Moreover, to ensure the function and vitality of the cells, these devices were equipped not only with a fluidic system that ensured the supply of culture medium, but also with systems for regulating temperature, humidity and gas atmosphere in the modules where the cells were incubated. A common feature of the apparatus of this first generation was that they were mono-parametric [4]. During the past 20 years these devices have found numerous applications in biology and medicine, for instance, in the study of ligand-receptor interactions, in the analysis of the action of chemicals and drugs on specific cell targets, in the assessment of the chemosensitivity of cancer cells, and many more [5–7].

These sensors were followed by a new generation of sensors characterized by the presence on the silicon chip of several sensor types, the so-called multi-sensor arrays, with which many parameters can be measured simultaneously: impedance (adhesion, cell shape), acidification or peri-cellular acidosis (metabolism) and oxygen consumption (respiration) [8–10]. From the very beginning, these new systems were developed with a view to their possible applications in research areas such as the chemosensitivity of tumor cells and drug screening. More recently, examples of successful applications in toxicology, pharmacology, and even in the field of ecology can be found in relevant literature [11–14].

With the application of multi-sensor technology arose the necessity for the development of methods of preparation of cells and tissues suitable for the investigation of such specimens in these new devices. At first, the use of permanent cell lines was imperative, because they are easier to handle in the sensor devices. But in the meantime, considerable experience has been acquired with primary and organotypic cultures, as well as tissue slices [15].

This paper describes the preparation and management of choroid plexus epithelial cells (CPC) on multi-sensor arrays as an *in vitro* cell model and presents results of how these cells react when treated with compounds influencing their metabolism and transport functions. The significance of choroid plexus lies in the fact that it is a major source of cerebrospinal fluid, which circulates through the brain ventricles and the subarachnoid space [16]. Because of the complex network of interactions in the whole body, appropriate cellular models under axenic conditions are required to understand the molecular basis of specific mechanisms of the choroid plexus epithelial cells, since they are the main cell element of this organ. These requirements were taken into account in the experimental approach described here with a resulting high relevance for cell biology in general and medical research in particular (neurology, neurosurgery, pharmacology, *etc*.).

## 2. Experimental Section

### 2.1. Cell Cultures

Choroid plexus of chicken embryo 14 ED embryos (20–30 embryos pro assay) were dissected and collected in culture medium (DMEM/F12 Gibco, supplemented with penicillin and streptomycin, L-glutamine, insulin, EGF, hydrocortisone, NEA, transferrin, 10% FCS). After dissection, the choroid plexus were dissociated with trypsin + EDTA (Gibco) in a Petri dish and using a magnetic stirrer at 37 °C in a CO_2_ incubator (Heraeus). Subsequently, the cell suspension was passed through a cell strainer (BD) and rinsed with medium with 10% FCS (to inactivate trypsin). The resulting cell suspension was centrifuged at 2,400 rpm for 4 min (cyto-centrifuge Sartorius), the supernatant discarded and the pellet re-suspended in fresh medium and the cells counted with Trypan Blue (Sigma-Aldrich). Subsequently, the chips were inoculated with 1.5 or 3 × 10^5^ cells each. Parallel to this, well plates with Thermanox slides (Nunc) were also seeded with a similar cell suspension (2 × 10^6^ pro well).

Although the chips are made of silicon, a material suitable for cell cultures, we coated their surfaces with collagen I, which considerably improves attachment conditions for the choroid plexus cells [17]. A solution of collagen I (20 μg/mL in 0.02 N acetic acid) was applied to the surfaces of the chips, which were then incubated at 37 °C for 1 h. Subsequently, they were washed with culture medium and used immediately or refrigerator-stored. Thermanox slides were coated following same protocol.

The cells were first cultured without cys-arabinoside (Sigma-Aldrich) on both chips and Thermanox slides. After 24 h the medium was replaced by fresh medium containing cys-arabinoside, a substance able to suppress the growth of fibroblasts and endothelial cells, but which does not affect the epithelial cells of the choroid plexus. After a culture period of 24 h or longer (until 70–80% of the chip surface was covered with choroid plexus cells) the chips were transferred to the sensor device. The measurements in the sensor device were conducted with a medium (low buffering capacity), which did not contain cys-arabinoside. The measurement times in the sensor unit ranged from 24 to 72 h, or even longer according to experiment requirements. The growth of cells on Thermanox plates was regularly monitored using an inverted microscope (Olympus) with phase contrast. As, in this case, the chips were opaque, the examination was performed by reflected light with a stereomicroscope (Wild, Heerbrugg, Switzerland).

### 2.2. Sensor Device

The Bionas 2500^®^ analyzing system (Bionas GmbH, Rostock, Germany) was used in these studies. A detailed description of this system has been published previously [13]. Briefly, the device is composed of six modules, which are connected with a pump and control units. The culture media, with or without drugs, first flow to and then through each module containing the sensor chips and are then directed to a waste collector. The reference electrode is placed in the fluidic pathway behind the sensor modules. The sensor chip (metabolic chip Bionas Discovery™ SC1000) used here, contains a sensor array composed of: (a) two Clark-type sensors, (b) five ISFETs (ionic-sensitive field-effect transistors) and (c) one IDES (inter-digitized electrode sensors). Such sensors allow simultaneous determination of cellular oxygen consumption (a), peri-cellular acidification (b) and cell impedance (c) in on-line regime and without any labeling.

Before the sensor analyzer is used, the tubes of the fluidic system have to be disinfected by perfusing them with 70% ethanol, followed by PBS and a final washing with culture medium, in our case DMEM/F12 (see above). The sensor chips should also be disinfected according to a similar protocol as above. The sensor chips with CPC cells (see above) were placed into the modules and maintained in the Bionas device at 37 °C.

The following operational parameters of the Bionas Analyzing System 2500^®^ were selected. The flow rate was 56 μL/min with an alternate cycle of “go” and “stop” phases of 4 min each. During the “go” phase choroid plexus cells attached to the chip received fresh medium with or without compounds (see below). During the “stop” phase oxygen consumption and extracellular acidification were continuously measured in the supernatant. Changes in the rates of these parameters were detected for each single stop phase, representing the metabolic activity of the cells at that time and are given as single data points in the diagrams.

The presence of cells in contact with IDES was detected and expressed in terms of cell impedance. These measurements provided information on the extent of cell adhesion to the substrate precisely at the IDES areas and were independent of pump activity. However, in consideration of the influence of interferences, the data point values during the “stop” phase were averaged. All data obtained have been presented normalized and were processed with Origin 8.0.

### 2.3. Compounds

To induce interpretable changes in metabolism and behavior of choroid plexus cells the following compounds were selected:

- Sodium fluoride (NaF, Merck) that dose-dependently inhibits glycolysis and anaerobic metabolism; in addition, direct effects of NaF on mitochondria have been described (see below)
- Potassium cyanide (KCN, Riedel-de-Haen) that inhibits cellular respiratory processes, especially at the level of mitochondria,
- Forskolin (generously provided by Prof. Dr. M. Diener, University of Giessen, Germany), an agonist of adenylate cyclase, an enzyme that stimulates and regulates the synthesis of cAMP,
- Acetazolamide (Sigma-Aldrich) that inhibits carbonic anhydrase, a key enzyme in water transport processes in the choroid plexus.

These compounds were implemented in the following concentrations: NaF 20 mM, KCN 5 mM and 10 mM, forskolin 5, 10 and 25 μM, acetazolamide 10 μM, 100 μM, 1 mM and 2 mM. The compounds were applied during periods of time ranging from 1 to 24 h, followed by a variable period, during which the cells received culture medium containing no compound, with the aim of detecting possible late effects and estimating the degree of cell recovery after a certain treatment.

At the end of each experiment, the chips were examined and photographed under a stereoscopic microscope, and then fixed chemically for electron microscopy. The Bionas device has six modules that were distributed as follows: two modules were destined for the controls, two for compound 1 and the remaining two for compound 2. This procedure facilitates comparative analysis. At least four chips per compound and concentration were measured and evaluated. Chips which created disturbances during experiments were excluded. The experiments were performed (per compound and concentration) on at least two different days.

### 2.4. Electron Microscopy

Chips and Thermanox slides were fixed in 3% glutaraldehyde (Polysciences Inc.) in PBS. For scanning electron microscopy in a FEI 30XL ESEM™ at 10 kV, specimens were post-fixed with osmium tetroxide, dehydrated in a series of alcohols, critical point dried and sputtered with platinum [18,19]. Transmission electron microscopy followed a protocol described elsewhere [20,21]. Briefly, fixation was performed with 3% glutaraldehyde in PBS, post-fixation with osmium tetroxide, dehydration in a series of alcohols and embedding in Embed 812 (Electron Microscopy Sciences, USA). Epoxy resin mixture was poured on the cells and polymerized at 60 °C. The polymerized resin layer with the cells can be easily removed from the silicon chip. Ultrathin sections (80 nm thick) were stained with uranyl acetate (Merck) and lead citrate (EMS, USA) and examined in a FEI Tecnai™ Biotwin 120 transmission electron microscope at 60 kV.

## 3. Results and Discussion

### 3.1. Cultures of Choroid Plexus Epithelial Cells (CPC)

**Figure 1.**
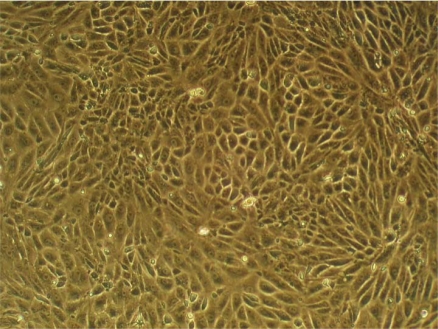
Phase contrast of choroid plexus cells grown on Thermanox for 4 days. The cells form a monolayer displaying a polygonal shape. The culture was previously treated with cys-arabinoside to eliminate cell contamination. 20X objective.

The preparation of the choroid plexus of the chick embryo was performed—with slight modifications—according to a protocol already described [22]. Following this protocol, we have achieved the formation of cell monolayers composed almost exclusively of choroid plexus epithelial cell (Figure 1). This was made possible by the addition of cys-arabinoside, an inhibitor of DNA synthesis, to the culture medium, [22]. The CPCs have a nucleotide transport system unable to uptake cys-arabinoside; therefore growth and morphology of these cells remain unchanged. In contrast, the other cells of plexus (fibroblasts, endothelia) take up the cytostatic, which prevents their proliferation and growth. The result is pure cultures of CPC.

When the cell suspension was applied to either chips or Thermanox slides, the cells attached at an early stage during the first 24 h, showing a variable shape, consistent with the process of attachment to substrate. After 2 days cultivation the cells began to aggregate with their congeners, constituting the monolayer 2 days later (Figure 1). Comparative studies with other coatings (laminin, fibronectin and collagen IV) have shown that CPCs attach faster on collagen I-coated surfaces, a circumstance beneficial to our studies with the sensors, as we can start the measurements earlier.

**Figure 2.**
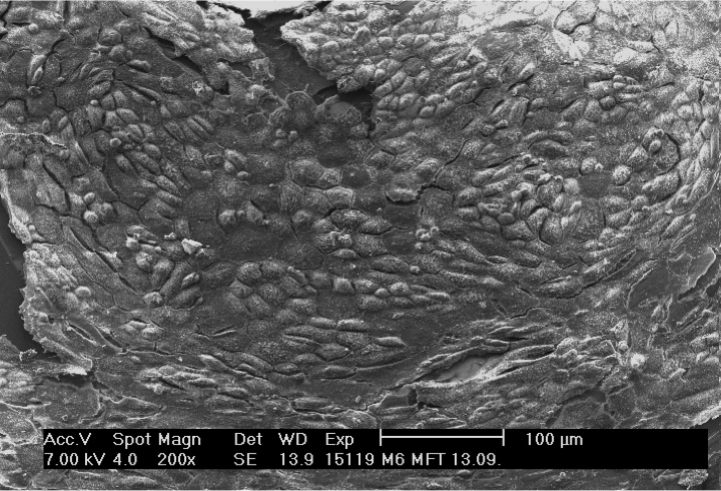
Scanning electron microscopy of CPCs growing on the sensor chip. The cells have numerous microvilli at the apical pole and show a marked relief in the central part that corresponds to the nucleus.

Electron microscopy revealed the surface ultrastructure of these monolayers and confirmed their specific cell composition (Figure 2). The cell borders were marked by numerous thin microvilli, giving the image a mosaic-like appearance. In the center of the cells, apical pole microvilli and cilia, typical attributes of these cells lost during enzymatic dissociation, reappeared. Transmission electron microscopy confirmed these diagnoses, and also enabled determination of the participation of cytoskeleton and cell membrane, forming intercellular contacts and anchorages to substrate (not shown). Cells grown on silicon chips or Thermanox displayed exactly the same patterns.

**Figure 3.**
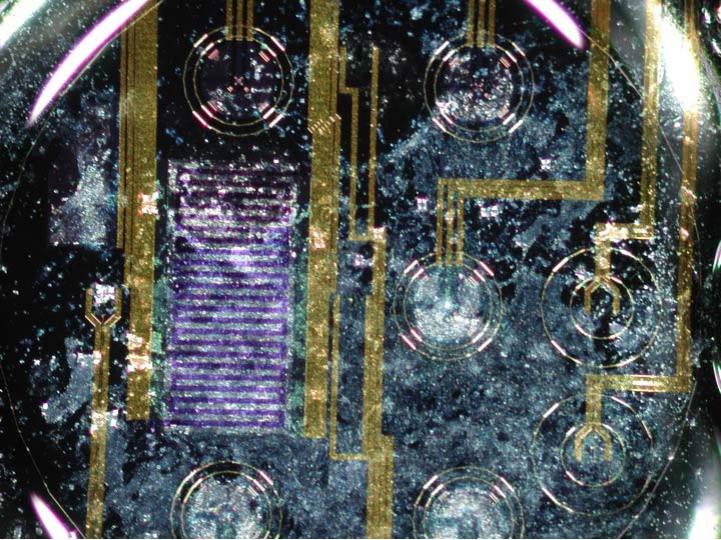
Scanning electron microscope image shows groups of cells arranged in monolayer growing on the chip. The CPCs can be seen as a grey veil covering the major part of the visible chip surface.

Before the silicon chips with the cells were placed into the sensor device, every chip was examined under a stereomicroscope to estimate the quality of the monolayers and their topographic relationship with the sensors (Figure 3).

### 3.2. Measurements under Experimental Conditions

**Figure 4.**
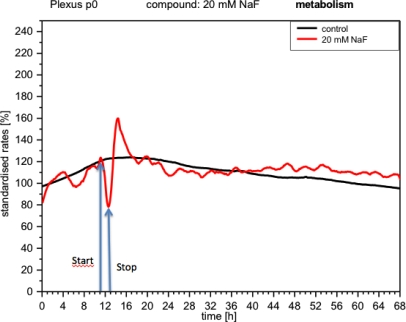
Online monitoring of metabolism of CPCs exposed to fluoride (details in the graph). Note the biphasic curve indicating a strong inhibition, followed by a similarly strong activation. Start and Stop: Beginning and end of compound application respectively.

CPC responded to NaF with changes in activity detectable with all three types of sensors (IDES, ISFET, Clark). The metabolism measurements (peri-cellular acidification or acidosis) showed that the curve dropped immediately to 80% (Figure 4). When the medium was replaced with fresh medium, the metabolism recovered very quickly (upward curve), reaching values high above the initial value (160%), stabilizing a few hours later and behaving like the control.

**Figure 5.**
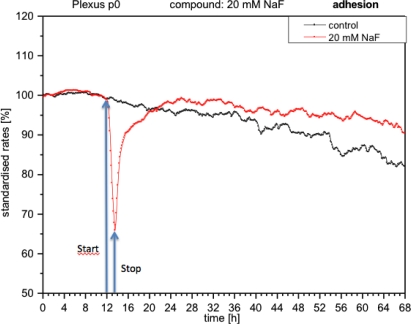
Online monitoring of cell adhesion of CPCs exposed to fluoride. The impedance decreased very quickly and recovery takes a long time to reach values similar to the controls.

With the presence of NaF at the module, the cells reacted with an immediate decrease in impedance (approximately 40%), which means that adhesion was reduced and consequently cells became detached (Figure 5). When the medium was replaced with fresh medium without NaF, the curve rose again, reaching the initial values prior to the application of fluoride. These measurements indicate that the cells had re-adhered.

**Figure 6.**
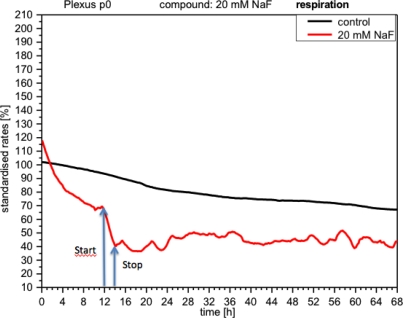
Online monitoring of respiration (oxygen consumption) of CPCs exposed to NaF. This parameter decreases, but does not recover completely (compare with the control).

NaF discretely inhibited cellular respiration (curve down by 30%), which, although it later recovered after the removal of NaF, still remained below the control levels and displayed a rather unstable profile (Figure 6).

**Figure 7.**
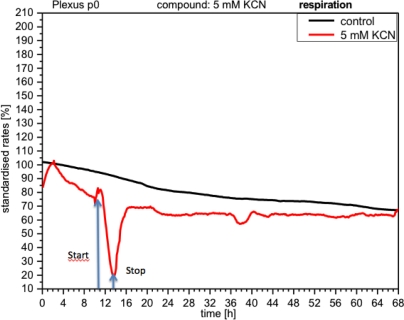
Online monitoring of respiration of CPCs exposed to cyanide. Note the well-known effect of cyanide on oxygen consumption, with recovery within a few hours.

**Figure 8.**
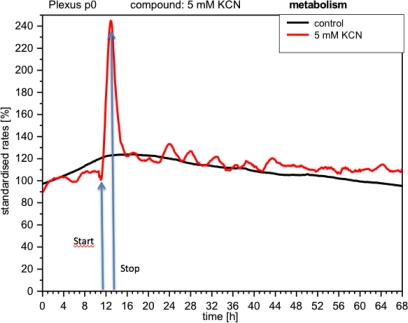
Online monitoring of metabolism of CPCs exposed to cyanide (same experiment as Figure 7). Notable activation of metabolism (acidification) observed in all experiments with cyanide.

**Figure 9.**
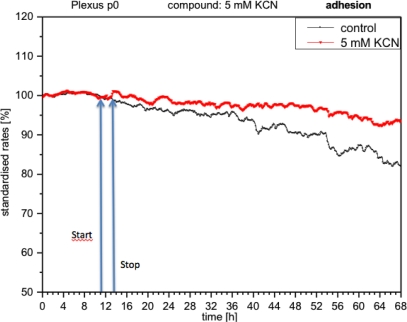
In contrast, adhesion of CPCs (impedance) does not show any changes (same type as experiment Figure 7).

CPC exposed to potassium cyanide (KCN) responded with a remarkable increase in metabolism (over 140%), *i.e.*, with higher peri-cellular acidification, which, after the removal of cyanide from the medium, reverted within a few hours to levels similar to control levels (Figure 7). Impedance remained unchanged under these conditions, indicating that no relevant changes in cell adhesion had taken place (Figure 8). By contrast, cellular respiration underwent a significant drop of approx. 60% (Figure 9). After removal of the cyanide, the cells recovered within a few hours, reaching levels close to the controls. This applies for both used concentrations of 5 and 10 mM of KCN.

The metabolic changes triggered by NaF are explained by the inhibition of enolase, a key enzyme in glycolysis [23], resulting in a reduction in the extrusion of protons (peri-cellular acidification) from the cell [3,6,15]. Moreover, it has been well-documented that NaF can induce apoptosis through metabolic stress, including changes in cell proteins, mitochondria and cell nucleus [24,25]. The lipid peroxidation that accompanied these processes diminished membrane fluidity, with the appearance of blebbing and detachment of the cells [26]. Such events are consistent with our impedance measurements. Finally, the changes in respiratory parameters could be due to the effect of NaF on the mitochondria as a result of impairment of the stability of their membranes through lipid peroxidation [27].

The experiments with KCN are interesting, as the increase in glycolysis observed simultaneously with the blocking of respiration could have a compensatory significance. The blocking of respiration does not appear to be lethal (under the conditions used here) because the ATP needed can be provided from the glycolysis, which appeared strongly activated in these experiments (increase of peri-cellular acidification) and thus cells can survive and recover [28,29]. Investigations carried out on hepatocytes and cell lines support this assumption [29].

**Figure 10.**
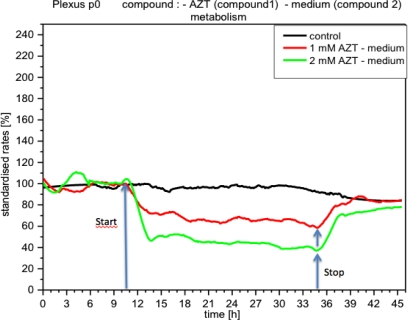
Online monitoring of metabolism of CPCs exposed to acetazolamide. The metabolic activity falls rapidly and remains quite stable as long as the compound is present. CPCs recover quickly when acetazolamide is removed. Note the differences of impedance depending on the compound concentration. The curves represent two different experiments.

The application of acetazolamide and forskolin triggered distinct responses from the plexus cells. Metabolism decreased immediately upon acetazolamide reaching the cells (Figure 10). Within a short time the reduction in metabolism reached values of up to 50%, remaining at this level as long as acetazolamide (2 mM) was present in the medium. With a concentration of 1 mM acetazolamide, metabolism was reduced by approx. 35%, whereas with 10 μM and 100 μM no concentration effects were detected.

**Figure 11.**
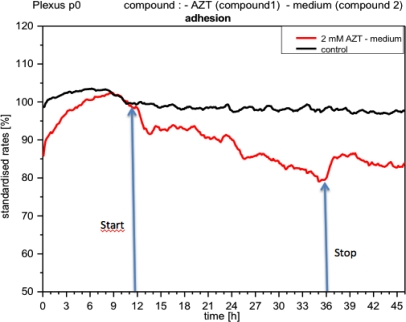
Online monitoring of adhesion of CPCs exposed to acetazolamide. The impedance diminishes progressively, with partial recovery after compound is removed.

Although metabolic activity recovered quickly upon the medium being replaced by a fresh one (without compound), it still did not reach the levels measured prior to application of the compound. Impedance, *i.e.*, cell adhesion, under the influence of acetazolamide, gradually diminished, and recovered after removal of the compound, but did not reach the control levels (Figure 11). Respiration, however, did not seem to change significantly (not shown).

The mechanism of action of this compound should be taken into consideration in the interpretation of these CPC responses. Acetazolamide inhibits carbonic anhydrase, an important enzyme for the functions of the choroid plexus and the formation of cerebrospinal fluid [30,31]. It has been established in a number of different cell types that peri-cellular acidification is dependent on carbonic anhydrase [32,33]. Therefore, inhibition of this enzyme would have to cause a decrease in acidification, which is precisely what we see in our experiments. As carbonic anhydrase is also located in mitochondria, the question arises as to whether acetazolamide can modify the respiration of the cells [33,34]. However, acetazolamide diffuses slowly into intact mitochondria, as this carbonic anhydrase is not very accessible to this compound [35]. These circumstances may explain our results on cell respiration and this compound. And finally, changes in adhesion were obvious, but not easy to interpret. Although studies with tumor cells in which the blockade of iso-enzymes of carbonic anhydrase (CA type IX located in trans-membranous position) alters cell adhesion [36], would, in a preliminary assessment, seem to support our results, additional investigations are nevertheless necessary to clarify this relationship.

**Figure 12.**
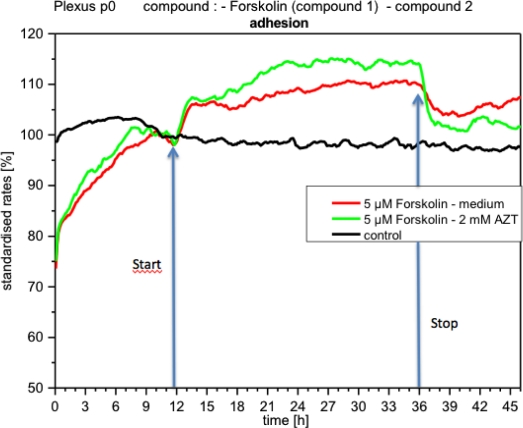
Online monitoring of adhesion of CPCs exposed to forskolin. The impedance dropped by approximately 5%, when it was removed and substituted by medium alone. When acetazolamide was applied after forskolin, impedance decreased clearly around 15%, remaining close to control levels. The curves are from two separate experiments.

Impedance made the response of CPC to forskolin evident (Figure 12). The increase of impedance indicates that cells adhered more strongly to substrate under forskolin than in the controls and, after approx. 2 h, the curve slowly rose and stabilized. In this context the question arose whether a consecutive application of acetazolamide and forskolin could trigger particular responses of CPCs, different to those obtained after a single application of these compounds. When forskolin was first applied, then removed and replaced by acetazolamide, the impedance dropped more rapidly than when it was replaced by medium alone (Figure 12). In contrast, in the sequence acetazolamide-forskolin no effects of forskolin on impedance were observed. Although this response was repeatedly observed, additional assays are required for a functional interpretation. The two other parameters, metabolism and respiration, did not change (not shown). Under the influence of forskolin metabolism behaves as in the controls. When, after forskolin, fresh medium was applied, the cells did not alter their activity. Respiration under forskolin did not show significant changes.

The changes described are consistent with the well-known properties of the diterpene forskolin. Forskolin activates adenylate cyclase, increasing intracellular cAMP [37]. It has been reported that an increase in intracellular cAMP levels increases cell adhesion and strongly inhibits motility [38]. These observations agree well with our findings on CPC, also an epithelial cell type.

However, interpretation of the activity patterns of metabolism and respiration are somewhat more complicated. On the one hand, forskolin appeared to stimulate the utilization of lactate in diverse cells and tissues [39]. But, on the other hand, forskolin significantly reduced the rate of glucose utilization (about 50 to 60%), apparently competing with the glucose transporter [39]. This could explain our measurements, in which no significant changes of metabolism were detected. Forskolin did not seem to affect the consumption of oxygen and this agrees with our observations [40].

However, in long-term experiments, forskolin is able to affect the biogenesis of mitochondria, increasing their number in adipocytes *in vitro* [41]. Whether these effects of forskolin are also relevant for CPC cultures has not been considered as yet in our studies.

## 4. Conclusions and Final Comments

This study demonstrates that is possible to produce hybrids of choroid plexus cells and silicon chips with sensors, which enable real-time measurement of dynamic events of a cell population *in vitro*. The dynamics of the cells are reflected in the selected parameters: acidification, oxygen consumption, and adhesion. The sensor device has proved to be very reliable and robust in both short- and long-term experiments.

Our studies revealed new aspects in the response of CPC to the compounds used. NaF acts on several cell targets and the simultaneous registration of cell responses allows correlations between the different parameters examined.

In the case of cyanide (KCN) it is noteworthy that, not only is cell respiration inhibited, a well-known effect of this compound, but at the same time, activation of glycolysis occurs. ATP produced by glycolysis can compensate KCN respiratory deficits and the cells survive. Acetazolamide is a drug that inhibits essential enzymes in the processes of water transport in the choroid plexus. Thus, the decrease of metabolism was predictable, whereas the changes observed in cell adhesion are rather novel. Whether the reduction of adhesion is related to attachment to substrate and cell-to-cell contact deserves additional investigation. These results correlated to data from the literature available, which demonstrate the presence of an isoform of CA located at the cell membrane. Forskolin was applied as a positive stimulus to the activity of the CPCs, the increase in adhesion confirming these expectations.

Finally, we would like to highlight properties of the sensor device used. It proved to be very flexible, offering a diverse menu of settings for the application of compounds, for example the possibility of sequential application of the same substance at different doses or different substances at different intervals (synergies or incompatibilities). These strategies are important in order to obtain a comprehensive and precise picture of the dynamic responses of a determined cell population or tissue *in vitro*. The use of sensor platforms in experimental medicine could have advantages, which should not be underestimated. Furthermore, it is a possible contribution towards the reduction of the number of studies with animals, particularly in diagnosis and drug development.
